# Effect of interactive, multimedia-based home-initiated education on preoperative anxiety inchildren and their parents: a single-center randomized controlled trial

**DOI:** 10.1186/s12871-023-02055-7

**Published:** 2023-03-28

**Authors:** Huiyan Hou, Xie Li, Yun’an Song, Yingying Ji, Menglian Sun, Dan Wang, Jiali Jiao, Jifang Qu, Hongbin Gu

**Affiliations:** 1 Department of Anesthesiology, Shanghai Children’s Medical Center, School of Medicine, Shanghai Jiao Tong University, Shanghai, P.R. China; 2 Department of Ophthalmology, Shanghai Children’s Medical Center, School of Medicine, Shanghai Jiao Tong University, Shanghai, P.R. China; 3 Institute of Translational Medicine, Shanghai Jiao Tong University, Shanghai, P.R. China; 4 Department of Anesthesiology, Fujian Children’s Hospital (Fujian Branch of Shanghai Children’s Medical Center), College of Clinical Medicine for Obstetrics & Gynecology and Pediatrics, Fujian Medical University, Fuzhou, P.R. China

**Keywords:** Preoperative anxiety, Children, Interactive, Multimedia-based, Parents

## Abstract

**Background:**

Anesthesiologists need to appreciate the impact of preoperative anxiety in children. The present study aimed to explore whether interactive multimedia-based home-initiated interventions could effectively relieve preoperative anxiety in pediatric patients.

**Methods:**

In this prospective study, we compared preoperative anxiety between two groups of children aged 4–9 years. Children in the control group received a question-and-answer (Q&A) introduction, and children in the intervention group received multimedia-based home-initiated preoperative education using comic booklets, videos, and coloring game books. Differences in anxiety between the two groups were evaluated by the modified Yale Preoperative Anxiety Scale-Short Form (mYPAS-SF) at four time points: in the ophthalmology outpatient clinic before intervention as the baseline (T0); in the preoperative waiting area (T1); at the time of separating from their parents and moving to the operating room (T2); and at the time of anesthesia induction (T3). Parental anxiety was assessed by the Self-rating Anxiety Scale (SAS) and Visual Analog Scale (VAS) at T0 and T2. Other related information was collected by questionnaire.

**Results:**

Eighty-four children who underwent pediatric strabismus in our center between November 2020 and July 2021 were included in this study. An intention-to-treat (ITT) analysis was performed on data from 78 enrolled children. Children in the intervention group exhibited lower m-YPAS-SF scores at T1, T2, and T3 than those in the control group (all p < 0.001). By using a mixed-effect model with repeated measurement (MMRM) after adjusting the m-YPAS score at T0 as a covariate, the interventional effect in terms of themYPAS-SF score was also significant over time (p < 0.001). The percentage of children with perfect induction compliance (ICC = 0) in the intervention group was significantly higher than that in the control group [18.4% vs. 7.5%], and poor induction compliance (ICC>4) was lower (2.6% vs. 17.5%, p = 0.048). The mean parental VAS score at T2 in the intervention group was significantly lower than that in the control group (p = 0.021).

**Conclusions:**

Interactive multimedia-based home-initiated intervention could reduce preoperative anxiety in children and improve the quality of anesthesia induction based on ICC scores, which may in turn impose a positive impact on parental anxiety.

**Supplementary Information:**

The online version contains supplementary material available at 10.1186/s12871-023-02055-7.

## Introduction

Preoperative anxiety in pediatric patients usually begins at admission when they know that they would undergo a surgical operation [1]. Up to 50–60% of children undergoing surgical procedures experience significant preoperative anxiety [2]. An unpleasant surgical or anesthesia experience, younger age, short preoperative preparation time, and parental anxiety are all risk factors for preoperative anxiety in children [3, 4]. Negative behaviors including separation anxiety, nightmares, aggression toward authorities, nocturnal enuresis, and eating disorders, can all increase preoperative anxiety in children, which also increases the postoperative analgesic requirements, prolongs the postoperative recovery process, induces emotional trauma in children and their parents, or affects the long-term cognitive and emotional development of children [5–7].

Pharmacological methods can often be used to address preoperative anxiety. Midazolam as a premedication is considered to be a reliable strategy for reducing preoperative anxiety [8]. However, it can be a stress source itself, and therefore strict compliance and administration timing are needed, especially in children, because it may cause high-level impulsiveness and delayed emergence from anesthesia [9, 10]. Non-pharmacological interventions are widely supported for use in reducing preoperative anxiety in children due to their advantage of improving children’s cooperation without causing adverse effects [11]. Studies have reported limitations of some strategies in reducing perioperative anxiety in children [12, 13]. For instance, transport in a ride-on toy car can relieve preoperative anxiety, but this measure is suitable and effective only for preschool children aged 2–5 years old [14]. Some researchers also suggest the use of clown doctors, video games, and other distraction tools to temporarily relieve anxiety in children, but whether these methods can help relieve the anxiety of their parents remains unknown [12]. Another study reported that parental presence at induction of anesthesia (PPIA) was ineffective in reducing the anxiety level of children [15, 16]; rather, it increased the heart rate and skin conductance level of the parents [17]. Given the large number of surgeries performed in children, it is imperative to optimize the surgical outcome by reducing perioperative anxiety in both children and their parents.

One of the interventional categories effective for reducing anxiety in children is by providing preoperative information to them in a manner appropriate to their developmental stage. The present study aimed to explore whether the use of comic booklets, videos, and coloring books for preoperative education could reduce anxiety in young pediatric patients.

## Participants and methods

This randomized controlled clinical trial was carried out from November 2020 to July 2021 upon approval from the Institutional Review Board of Shanghai Children’s Medical Center on 17/06/2020 (SCMCIRB-Y2020095), and was registered at the Chinese Trial Registry (ChiCTR2000039622) on 03/11/2020. Informed consent was obtained from the parents or legal guardians ofall participants. The trial was carried out following the Declaration of Helsinki, and the authors guaranteed the accuracy and completeness of the data and analysis of this paper.

## Participants

Children who underwent strabismus surgery at Shanghai Children’s Medical Center were recruited in this study. The inclusion criteria were children of either sex aged 4–9 years old with the American Society of Anesthesiologists (ASA) Physical Status I-II. The exclusion criteria were children with a previous history of surgery, developmental delay and verbal communication problems, hearing impairment or a history of mental illness, and who refused to participate in this study. The termination criteria were children or guardians who failed to cooperate with medical measures or withdrew from the study due to various unexpected events such as family changes or missing pets.

## Randomization, assignment, and blinding

The study used a block randomization method with a block length of four. Eligible children (and their parents) were equally randomized to a control group and an intervention group by using a random assignment table (with randomization information including random seed, length, and the number of blocks) generated by an independent statistical professional based on the block randomization methods. Patient assessment and data collection were completed by a trained full-time research nurse. The investigators who implemented the intervention based on randomized information and performed all the assessments and the statistical analysts were blinded to the grouping information.

## Preoperative education and the anti-anxiety method

After making the appointment for strabismus surgery in the ophthalmology clinic, all participating children and their parents were given preoperative education about hospital admission, preoperative preparation, fasting, anesthesia procedures and pain management, strabismus surgery, and postoperative recovery procedures.

## The control group

The children and their parents in the control group received a question-and-answer (Q&A) introduction to preoperative education from the researchers lasting for approximately 15–30 min, and no other preoperative education was given during the period before surgery.e.

## The intervention group

**Fig. 1:**
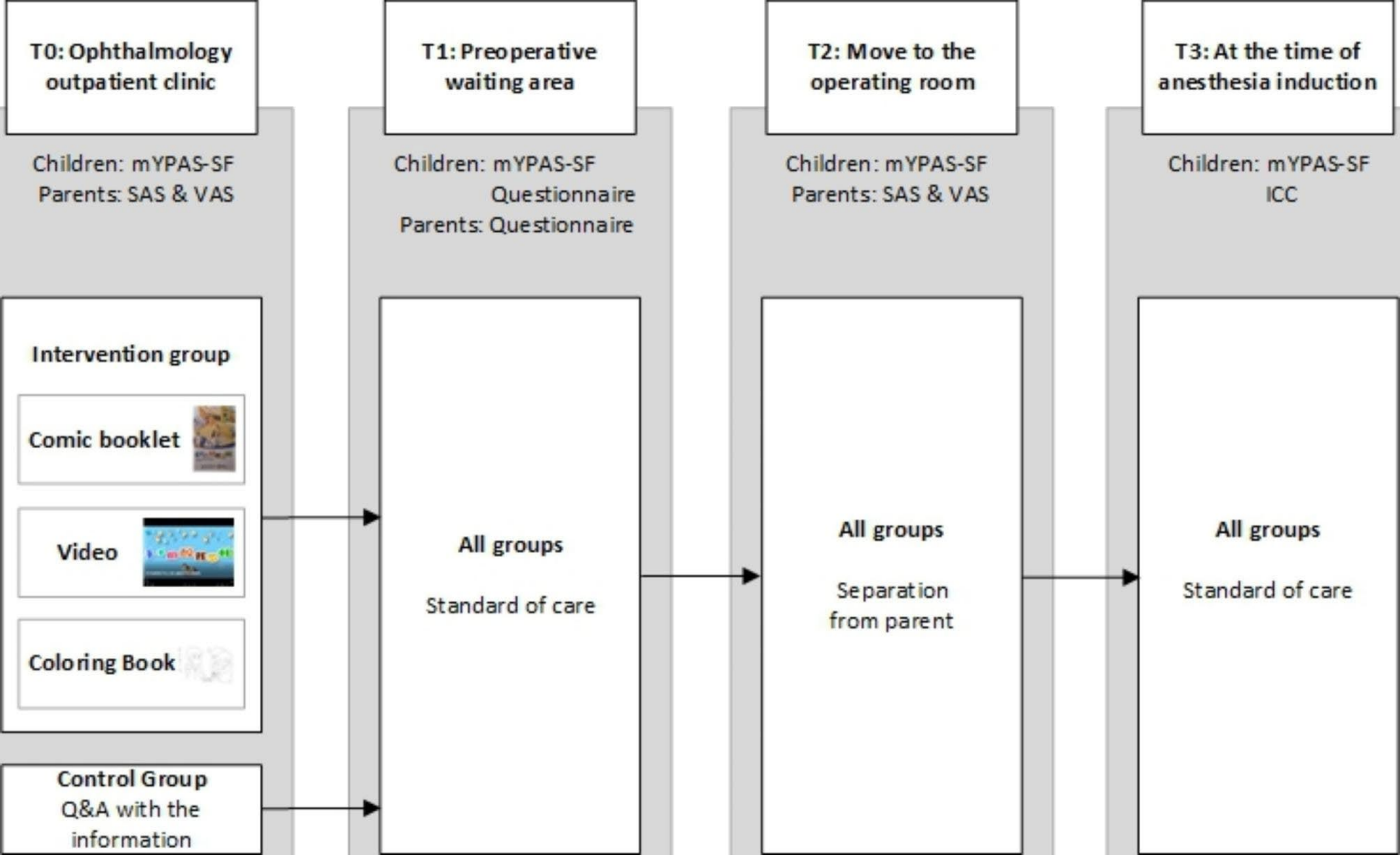
Study flow diagram. Note: Children in the intervention group received preoperative educational information through a comic booklet, video, and coloring game book, and children in the control group received preoperative educational information by Q & A. mYPAS-SF, modified Yale Preoperative Anxiety Scale-Short Form; SAS, Self-rating Anxiety Scale; VAS, Visual Analog Scale; ICC, The Induction Compliance Checklist

**Fig. 2:**
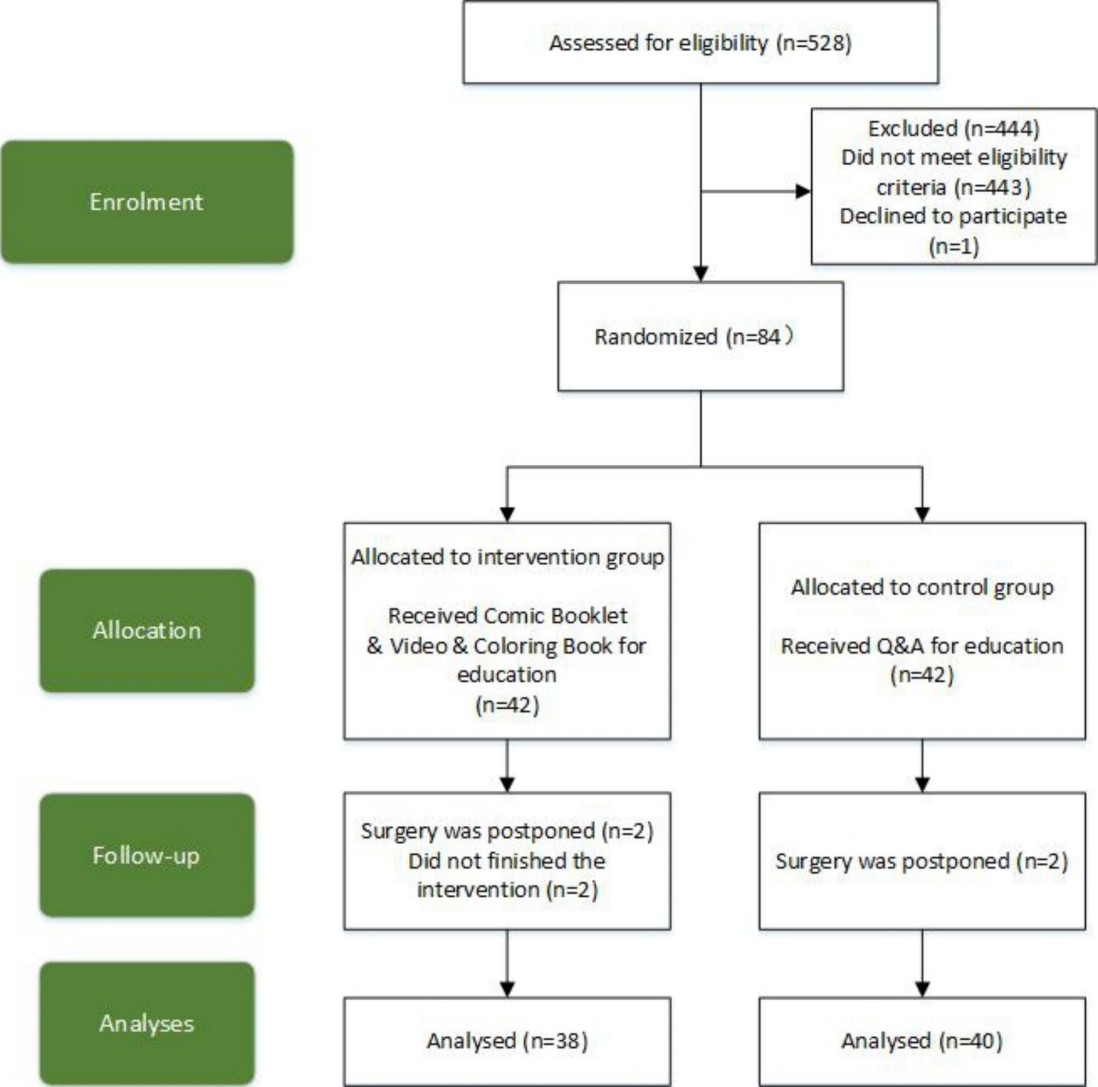
Flow diagram of the patients

The researchers prepared the preoperative educational information in the form of a comic booklet (S-Fig. 1), a video (S-Video), and an interactive coloring book (S-Fig. 2). The comic booklet was entitled “I’m not afraid of surgery and anesthesia”, which included admission registration, preoperative preparation, fasting, anesthesia procedures, pain management, and post-anesthesia recovery procedures. The children were allowed to choose the intervention method according to their interests, and the researchers made the content of the comic booklet into a ten-minute animated video entitled “I’m not afraid of surgery and anesthesia”. The interactive coloring game book covered admission, preoperative examination, strabismus surgery, and recovery procedures.

The first intervention stage was implemented in the ophthalmology clinic. The researchers distributed the comic booklets to the children and their parents, guided them to read the comic booklets, and then showed them the video. After that, they were given interactive coloring books to read while researchers gave them explanations about the surgical and recovery procedures, and this intervention lasted for aproximately 15–30 min.

The second intervention stage was implemented before hospital admission, during which the children and their parents were required to read the comic booklets or watch the video at home at least twice. The intervention was considered a success when the children and their parents read the comic booklets and/or watched the video at least twice and the children completed the interactive coloring book games; otherwise, the intervention was considered a failure and the cases involved were excluded from the study.

## Measures

The anxiety level of the children was scored by using the modified Yale Preoperative Anxiety Scale-Short-Form (mYPAS-SF) [18] by the blinded research nurse at four different time points: (1) in the Ophthalmology outpatient clinic before intervention as the baseline (T0); (2) in the preoperative waiting area (T1); (3) at the time of separating from their parents and moving to the operation room (T2); and (4) at the time of anesthesia induction (T3) (Fig. 1). mYPAS-SF is a simplified version of mYPAS, originally developed by Kain et al. [19]. The scale contains 18 items in four dimensions (activity, emotional expressivity, state of arousal, and vocalization), and the score range is 22.92–100.

According to the children’s behavior at T3, the Induction Compliance Checklist (ICC) was used to evaluate compliance by the researcher. Eleven items were rated as poor (ICC > 4), moderate (ICC = 1–4), or perfect (ICC = 0) [20].

Parental anxiety at T0 and T2 was measured by the SAS [21], which includes 20 questions (the higher the score, the higher the anxiety level), and the visual analog scale (VAS), which ranges from 1 (no anxiety) to 10 (extreme anxiety) [22].

The primary outcome variablewas the children’s anxiety level, scored by the mYPAS-SF at T3. The secondary outcome variables included children’s anxiety at other time points, children’s induction compliance, and parental anxiety levels. Other basic information and intervention completion data were collected at T1 from children and parents through questionnaires.

## Sample size estimation

According to the results of several existing studies in the literature and a preliminary study in our center [1], the mean mYPAS-SF score at T3 was approximately 60 ± 18 in the control group. With a two-sided significance level of 5% and an efficacy of 80%, 36 participants per group were required to detect a 20% decrease between the intervention and control groups (12 on the rating scale). The estimated rate of missed visits was 10%, and 84 participants were finally recruited.

### Statistical analysis

Data were analyzed with the ITT population. Normality was analyzed by the Shapiro-Wilk test. Data of normal distribution are reported as the mean ± standard deviation (SD) and compared with the Student’s t-test. Non-normally distributed data or ordinal data are presented as the median (interquartile range) and were compared with the Mann–Whitney U test. Categorical data are presented as numbers (percentages) and compared with the χ2 test or Fisher’s exact test. Changes in mYPAS-SF over time were analyzed using a mixed-effect model with repeated measurement (MMRM) analysis using m-YPAS scores at all follow-up time points (T1, T2, and T3) as the dependent variable, treatment as the main factor, m-YPAS scores at T0 as a covariate, and a random intercept to model within-subject correlation. A p value < 0.05 was considered statistically significant. R for Windows version 4.1.2 was used for statistical analysis.

## Results

### Demographic and clinical characteristics

Between November 2020 and June 2021, 528 children and their parents were screened for this study. Of them, 443 did not meet the inclusion criteria, one declined to participate, and 84 were enrolled, with 42 in each group. Of the 84 included children, the operation was postponed beyond the study period in four children due to personal reasons, intervention failed in the other two children, and the remaining 78 (40 in the control group and 38 in the intervention group) were available for analysis. The flow chart of the study in terms of patient enrollment, allocation, follow-up observation, and analysis is shown in Fig. 2. There was no significant difference in the baseline sociodemographic and clinical variables between the two groups (Table 1).

**Table 1:** Baseline characteristics of the children and their parents

Characteristics	Control(n = 40)	Intervention(n = 38)	P value
**Children’s characteristics**			
Age, yr	6.88 ± 1.28	6.66 ± 1.0	0.387
Age range, yr	4.08-9	4.75–8.42	
Female, n (%)	21 (52.5)	22 (57.89)	0.632
Male, n (%)	19 (47.5)	16 (42.11)	
Height, cm	122.9 ± 8.13	122.16 ± 6.76	0.663
Weight, kg	24.42 ± 4.64	24.23 ± 4.18	0.849
Body mass index, kg m^− 2^	16.08 ± 2.02	16.14 ± 1.68	0.895
Time interval between education to admission, day	6.32 ± 2.59	6.45 ± 2.52	0.833
Unilateral surgical cite, n (%)	14 (35)	12 (31.58)	0.749
Bilateral surgical cites, n (%)	26 (65)	26 (68.42)	
**Parental age range**			0.879
20–30, yr, n (%)	7 (17.5)	6 (15.79)	
30–40, yr, n (%)	25 (62.5)	22 (57.90)	
40–50, yr, n (%)	6 (15)	9 (26.31)	
>50, yr, n (%)	2 (5)	1 (2.63)	
**Parental education**			0.871
Primary school, n (%)	3 (7.5)	1(2.63)	
Junior high, n (%)	2(5)	4(10.53)	
High school, n (%)	7(17.5)	8 (21.05)	
Junior college, n (%)	13 (32.5)	12 (31.58)	
University, n (%)	15 (37.5)	13(34.21)	

Note: Age, height, weight, body mass index, and the time interval between education and admission were tested and validated to follow a normal distribution.Data was presented as the mean ± SD for continuous variables, and n (%) for categorical variables. P value calculation: Continuous variables were tested by two independent samples t-tests, and categorical variables were tested by chi-square tests

## Children’s anxiety and induction compliance

There was no significant difference in the baseline m-YPAS-SF scores at T0 between the control group and the intervention group (41.68 [31.26, 45.85] vs. 41.68 [37.51, 45.85], p = 0.859). The mean m-YPAS-SF score in the intervention group was significantly lower than that in the control group at T1, T2, and T3 (all p < 0.001). By using MMRM after adjusting m-YPAS scores at T0 as a covariate, the intervention effect in terms of the mYPAS-SF score was also significant over time (p < 0.001). The percentage of children with perfect induction compliance (ICC = 0) in the intervention group was higher than that in the control group [18.4% vs. 7.5%], and poor induction compliance (ICC>4) was lower (2.6% vs. 17.5%, p = 0.048) (Table 2).

**Table 2:** Children’s anxiety and induction compliance

Endpoints	Time/Scale	Control (N = 40)	Intervention (N = 38)	P value
mYPAS (median [IQR])	T0	41.68 [31.26, 45.85]	41.68 [37.51, 45.85]	0.859
	T1	31.26 [27.09, 41.68]	22.92 [22.92, 27.09]	< 0.001
	T2	55.22 [41.68, 62.50]	32.30 [22.92, 37.51]	< 0.001
	T3	60.43 [45.85, 72.93]	37.51 [31.26, 49.49]	< 0.001
Compliance Scale, n (%)	poor	7 (17.5)	1 (2.6)	0.048
	moderate	30 (75)	30 (78.9)	
	perfect	3 (7.5)	7 (18.4)	

Notes: mYPAS-SF: the modified Yale Preoperative Anxiety Scale-Short-Form; Compliance Scale: According to the child’s behavior at the time of placing the mask, their compliance during induction was classified as poor (ICC > 4], moderate (ICC = 1–4), and perfect (ICC = 0). T0 = baseline, in an ophthalmology outpatient clinic, before any intervention; T1 = in the preoperative waiting area; T2 = at the time of separation from parents, move to OR; T3 = at the time of anesthesia induction. P value calculation: Continuous variables were tested by two independent samples t-tests

**Fig. 3:**
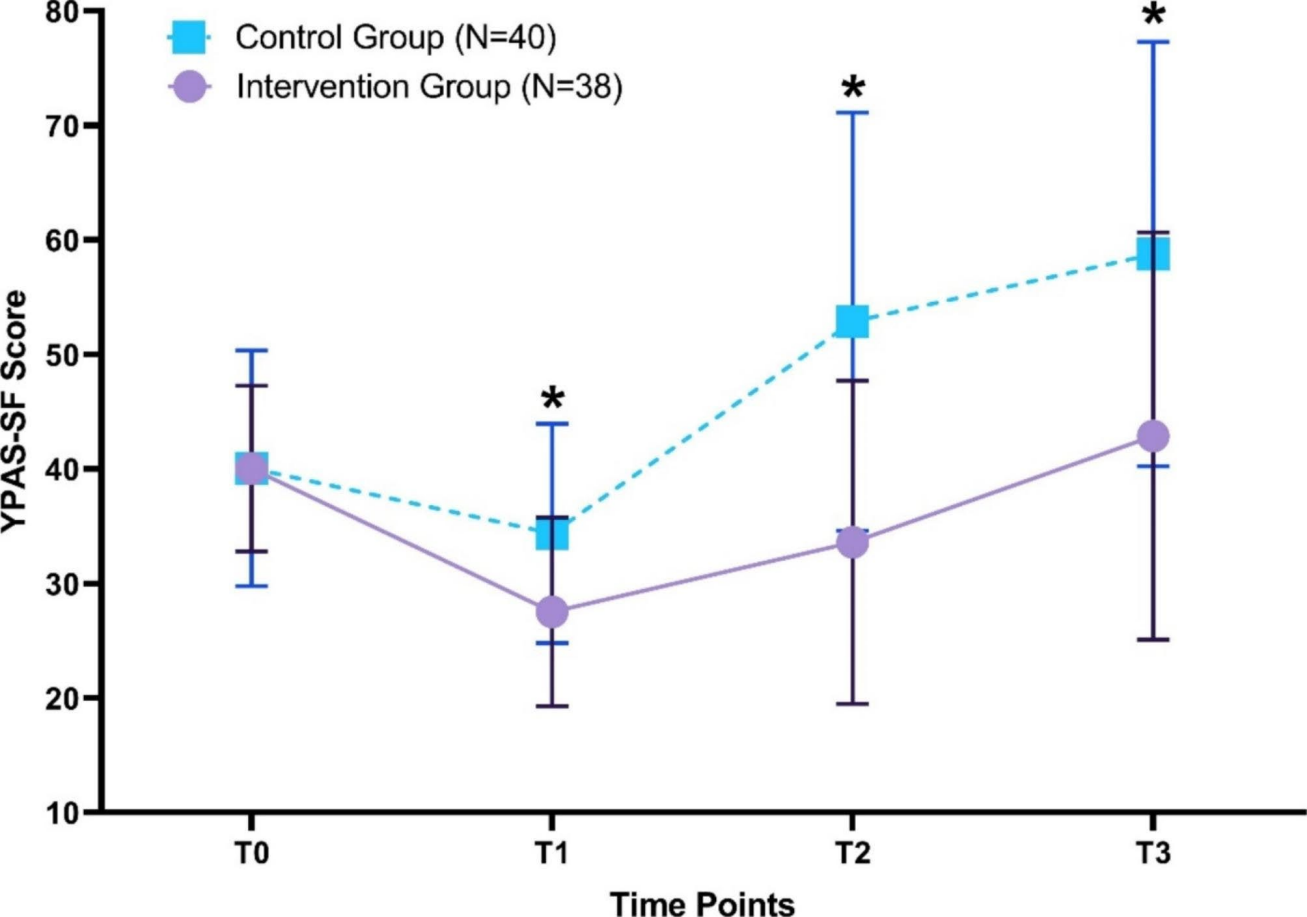
Trends of children’s anxiety. Note: mYPAS-SF: the modified Yale Preoperative Anxiety Scale-Short-Form; T0 = baseline, in the ophthalmology outpatient clinic before any intervention; T1 = in the preoperative waiting area; T2 = at the time of separating from the parents and moving to the operating room; T3 = at the time of anesthesia induction. Mixed effect analysis, group comparisons *p < 0.001

As shown in Fig. 3, the anxiety level in the intervention group was significantly lower than that in the control group at T1, T2, and T3 (all P < 0.001). The anxiety level was the lowest at T1 in both groups, and the anxiety level reached the highest at T3 in the control group.

## Parentalanxiety level

There was no significant difference in the mean VAS score and SAS score at T0 between the two groups. The mean VAS score at T2 in the intervention group was significantly lower than that in the control group (4.0 and 2.3 vs. 5.2 and 2.1, 95% CI: -1.2 (-2.20, -0.20), P = 0.021). The difference in the mean SAS score at T2 was not significant between the two groups (36.51 and 9.65 vs. 37.86 and 11.04, 95% CI: -1.34 (-6.02, 3.32), P = 0.568) (Table 3).

**Table 3:** Parental anxiety level

Endpoints	Time/Scale	Control (N = 40)	Intervention (N = 38)	P value
SAS (mean (SD))	T0	37.24 (10.44)	37.63 (9.39)	0.864
	T2	37.86 (11.04)	36.51 (9.65)	0.568
VAS (mean (SD))	T0	4.4(1.5)	4.7 (2.1)	0.573
	T2	5.2(2.1)	4.0 (2.3)	0.021

Note: SAS: Self-rating Anxiety Scale; VAS: anxiety Visual Analog Scale; T0 = baseline, in the ophthalmology outpatient clinic before any intervention; T2 = at the time of separating from their parents and moving to the operating room. P value calculation: Continuous variables were tested by two independent samples t-tests

## Evaluation of interventions

The evaluation of the intervention completion surveys and the information received from the children through questionnaires are shown in Table 4. Of the 40 children in the intervention group, 38 (95%) completed the intervention, and the remaining two (5%) failed the intervention, including one who neither finished the coloring book, watched the video, nor read the comic book, and the other one who did not complete the intervention because he did not like the coloring book.

**Table 4:** Evaluation of interventions by children

Evaluation of intervention	Completion Survey		Children(N = 40)	
Success (n = 38)	Have read the comic booklet once, watched the video once, and completed the coloring book	30		
	Have read the comic booklet once, watched the video twice, andcompleted the coloring book	5		
	Have read the comic booklet twice and finished the coloring book	0		
	Have watched the video twice and completed the coloring book	3		
Failure (n = 2)	Did not read the comic booklet, watch the video orcomplete the coloring book	1		
	Have read the comic booklet and watched the video but did not complete the coloring book	1		

Note: n, the number of children who answered yes to the question; N, the total number of children for whom the answer to the question was not missing

## Discussion

In this study, we investigated an approach that integrated several previous relatively independent approaches to provide children with an active participatory [23–26], multimedia-based home-initiated educational intervention prior to admission. Since this experiment incorporates parents and includes three aspects of children’s audio-visual stimulation, active painting, and reading, it is more effective than other experiments. We found that the anxiety level in children in the intervention group was significantly lower than that in children in the control group at all designated time points (during preoperative waiting, at the time of separation from their parents, and at the time of induction of anesthesia).During anesthesia induction, children in the control group had higher levels of anxiety and were less cooperative. It was also found that the parents of children in the intervention group had a lower VAS anxiety score when they were separated from their children. These results can serve as a complement to the literature concerning non-pharmacological approaches for the prevention and treatment of preoperative anxiety in pediatric patients, including education, behavioral techniques, PPIA, and complementary and alternative medicine (CAM) techniques [11].

The strengths of this study include the randomized controlled design, utility, and use of existing and validated behavioral-based scales to reduce observer bias. A similar study [26] showed that reading educational comic leaflets approximately one week before surgery could effectively alleviate anxiety levels in children aged 6–17 years old, but they did not address the effect on parental anxiety. It is commonly believed that children’s preoperative anxiety positively correlates with parental anxiety [27]. We did not perform a related analysis, but we found that parental anxiety scores measured by the SAS and VAS were inconsistent. The possible reason is that SAS is a clinical tool to analyze the subjective anxiety of the client, which evaluates the client’s emotional state over a period, while VAS measures the immediate emotional feeling. Li et al. [27]observed how parental anxiety influenced children to feel more frightened and be less cooperative. Thus, efforts should be made to decrease parents’ preoperative stress behaviorally or by using other interventions that could reduce their children’s anxiety [3]. Another study performed by Hilly et al. [28] reported that one-hour study two weeks before theoperation could reduce preoperative anxiety or even prevent the occurrence of postoperative adverse behavioral changes in children. However, this strategy often requires extra time and additional labor for support, while our approach is simpler and more effective. Our study found that preoperative education could effectively reduce preoperative anxiety in children. Kim et al. [29] also showed that the use of handheld tablet devices with interactive functions may be the most effective intervention strategy for reducing preoperative anxiety in children, which is consistent with our findings. More importantly, our broad inclusion criteria and uniformity of case selection may add credibility to the study and therefore be more applicable to pediatric patients with ophthalmological diseases.

Children older than four years old usually have already had a more developed sense of self and potential harm [8]. They are also better able to cooperate. In children, the perception of anxiety depends on the developmental stage and cognitive potential, and different responses can be observed among those facing the same stressor agent [3]. Studies have shown that most children who undergo surgical procedures wish to know more detailed information about what they will encounter and what will happen in the operating room. They usually prefer to have comprehensive information concerning their surgery, including information about pain, anesthesia, perioperative procedures, and potential complications [30]. Additionally, the more anxious the children feel, the more eagerly they want to know information about pain [30]. Children’s processing of preparatory information may be affected by multiple factors, including previous experience, developmental level, and comprehension [2]. The timingof providing preparatory information may also influence how much can be retained, although the optimum timing is not known. Some earlier work suggests that information should be given at least five days in advance for children aged six years or older and no more than a week in advance for children below six years of age [11]. From this point of view, proactive anxiolytic measures should be taken early even before hospital admission [11], so the intervention in our study was initiated in the outpatient clinic before admission to the hospital.

The results of our questionnaire investigation demonstrated that the intervention failed in a four-year-old boy who could fully understand the comic booklet because his parent interfered too much while watching the video, so he refused to engage with the coloring book. The other failure occurred in a seven-year-old boy because he did not like the coloring game, although he did well in reading the comic booklet and watching the animated video, and behaved cooperatively throughout the operation period. This interactive multidimensional early intervention provided additional help to pediatric patients, and effectively reduced their preoperative anxiety, which is consistent with our previous expectations.

Although there have been numerous clinical studies on how to reduce preoperative anxiety in children and a series of measures have also been proposed [12], specific factors affecting preoperative anxiety inchildren of different age groups are still not fully understood. For this reason, there are limited targeted interventions with controversial outcomes. The Home-Initiated-Programme-to-Prepare-for-Operation (HIPPO) intervention did not achieve any significant effect in diminishing children’s anxiety before an operation [31].Eijler et al. also reported that the provision of virtual reality exposure as a form of distraction therapy for children undergoing day surgery had no significant beneficial effect on anxiety and pain [32]. Fincher et al. instituted comprehensive play interventions but found no alleviation in perioperative anxiety in children [33]. The discrepancy may be due to the multifactorial etiology of preoperative anxiety in pediatric patients. Therefore, it is increasingly recognized that addressing preoperative anxiety should be a multimodal effort.

It was also found in our study that the anxiety level was the lowest at T1 in the preoperative waiting area in children of both groups, which was lower than the level at T0 in the same group. For this reason, we transformed the preoperative waiting area into a children’s play area where parents can accompany their children to play with toys, read picture books, watch cartoons, and play games with other children awaiting surgery. As a result, both children and their parents feel more relaxed and less anxious. In contrast, children in the control group all became vigilant when they were about to enter the operating room and separate from their parents and their anxiety level peaked at the time of anesthesia induction. This phenomenon shows that simple distraction does not solve the source of anxiety in children, while interactive and multidimensional preoperative education is the key to relieving children’s anxiety.

## Limitations

There are some limitations in this study. First, our multimedia products including comic booklets, videos, and coloring books were created based on the environment of our hospital and are personalized for children who come to the hospital for treatment and surgery, and thus may not be suitable for use in other hospitals. For instance, decorations and culture of our hospital are different from those of other hospitals. However, at any rate, our experience and practice may serve as a useful reference. In addition, we excluded children younger than four years old because they may not be able to read and write, so more studies are required to explore how to relieve anxiety in children younger than four-year old. Finally, we did not follow up with the children when they were returned to the ward, so it is unclear whether these measures can also help reduce postoperative maladaptive behaviors over time.

## Conclusions

The results of this practical randomized controlled trial demonstrated that participatory, multimedia-based home-initiated preoperative intervention could effectively relieve preoperative anxiety in children and reduce parental anxiety. It is an effective and noninvasive way to treat anxiety in pediatric patients.

## Electronic supplementary material

Below is the link to the electronic supplementary material.

Supplementary Material 1

## Data Availability

The datasets used and/or analysed during the current study are available from the corresponding author on reasonable request.
