# Supplementary material for: Effect of interactive, multimedia-based home-initiated education on preoperative anxiety inchildren and their parents: a single-center randomized controlled trial
Source: BMC Anesthesiol. 2023 Mar 28;23:95. doi: 10.1186/s12871-023-02055-7 (PMC10045252; doi:10.1186/s12871-023-02055-7)
Supplement: Supplementary file 1 — Supplementary Material 1 [file 12871_2023_2055_MOESM1_ESM.docx]

**S-Fig.1 I’m not afraid of surgery and anesthesia**


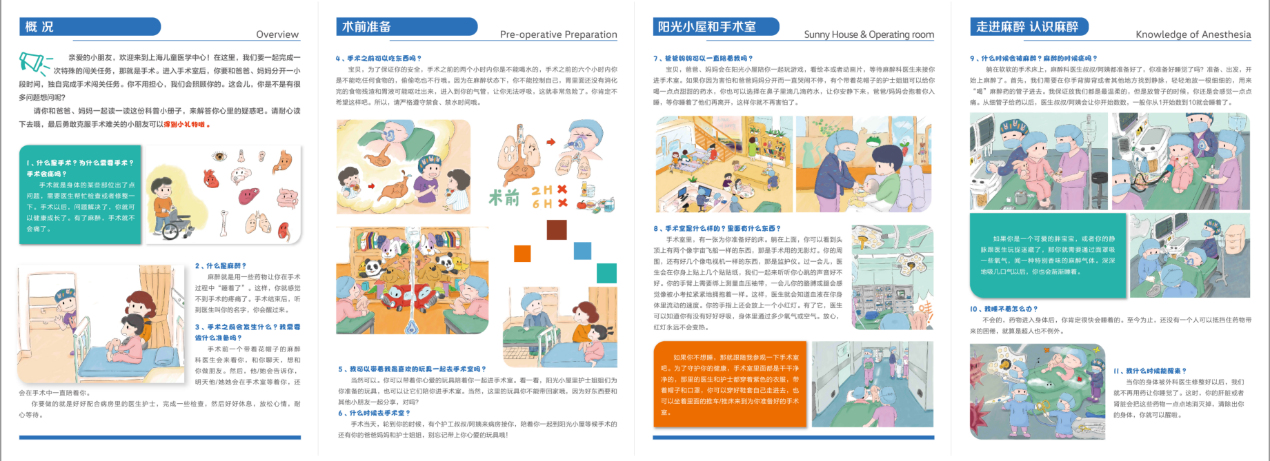


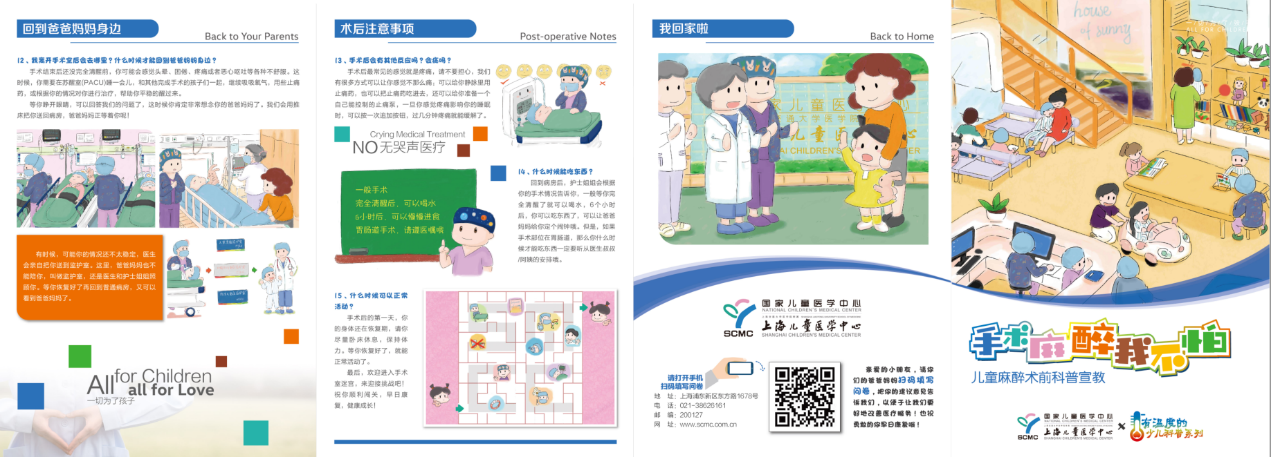


The comic booklet was entitled “I’m not afraid of surgery and anesthesia”, which included admission registration, preoperative preparation, fasting, anesthesia procedures, pain management, and post-anesthesia recovery procedures.

**S-Video link:**

https://www.bilibili.com/video/BV1Da411L78F?share_medium=iphone&share_plat=ios&share_source=WEIXIN&share_tag=s_i&timestamp=1655189592&unique_k=at96uY5

**S-Fig.2 Interaction coloring gamebooks**

**
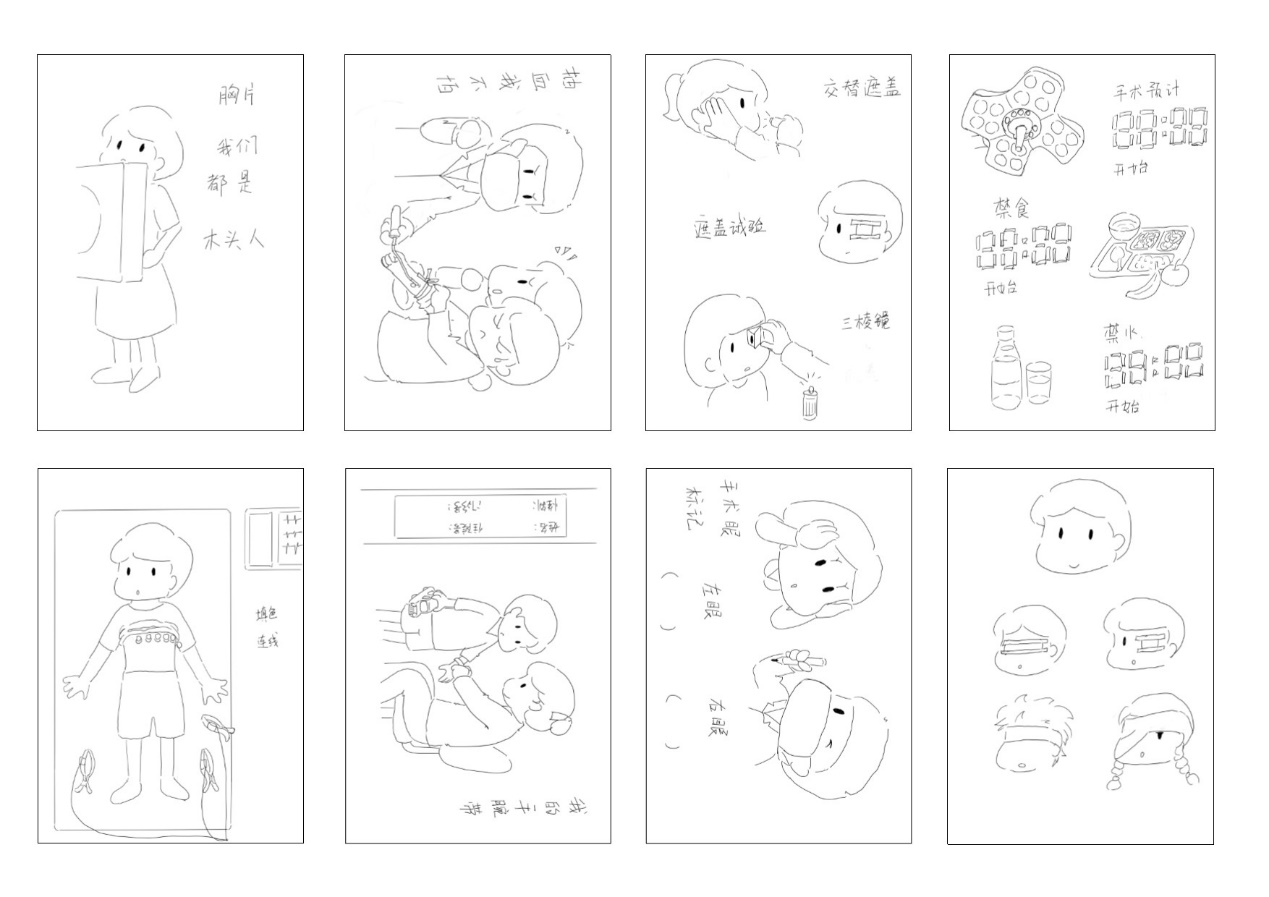
**

The interactive coloring game book covered admission, preoperative examination, strabismus surgery and recovery procedures.
